# Novel Molecular Tumor Cell Markers in Regional Lymph Nodes and Blood Samples from Patients Undergoing Surgery for Non-Small Cell Lung Cancer

**DOI:** 10.1371/journal.pone.0062153

**Published:** 2013-05-03

**Authors:** Oddmund Nordgård, Gurpartap Singh, Steinar Solberg, Lars Jørgensen, Ann Rita Halvorsen, Rune Smaaland, Odd Terje Brustugun, Åslaug Helland

**Affiliations:** 1 Department of Hematology and Oncology, Stavanger University Hospital, Stavanger, Norway; 2 Department of Cardiothoracic Surgery, Oslo University Hospital - Rikshospitalet, Oslo, Norway; 3 Department of Genetics, Oslo University Hospital - Norwegian Radium Hospital, Oslo, Norway; 4 Department of Oncology, Oslo University Hospital - Norwegian Radium Hospital, Oslo, Norway; University of Barcelona, Spain

## Abstract

**Introduction:**

Recent evidence suggests that microscopic lymph node metastases and circulating tumor cells may have clinical importance in lung cancer. The purpose of this study was to identify new molecular markers for tumor cells in regional lymph nodes (LNs) and peripheral blood (PB) from patients with non-small cell lung cancer (NSCLC).

**Methods:**

Candidate markers were selected based on digital transcript profiling and previous literature. *KRT19*, *CEACAM5*, *EPCAM, DSG3, SFTPA, SFTPC* and *SFTPB* mRNA levels were initially validated by real-time reverse transcription PCR-based quantification in 16 NSCLC tumors and 22 LNs and 12 PB samples from individuals without known cancer. Five of the candidate markers were selected for secondary validation by quantification in parallel tumor biopsies, regional LNs and PB samples from 55 patients undergoing surgery for NSCLC. LN and PB marker status were compared to clinicopathological patient data.

**Results:**

All selected markers except DSG3 were present at high levels in the primary tumors and at very low or non-detectable levels in normal LNs and PB in the first round of validation, indicating a potential for detecting tumor cells in NSCLC patients. The expression profiles of *KRT19*, *CEACAM5, DSG3, SFTPA* and *SFTPC* mRNA were confirmed in the larger group during the secondary validation. Using the highest normal LN level of each marker as threshold, 39 (71%) of the 55 patients had elevated levels of at least one marker in regional LNs. Similarly, 26 (47%) patients had elevated levels of at least one marker in PB. A significantly higher number of patients with adenocarcinomas had positive LN status for these markers, compared with other histological types (P = 0.004).

**Conclusions:**

Several promising molecular tumor cell markers in regional LNs and PB were identified, including the new *SFTPA* and *SFTPC* mRNAs. Clinical follow-up in a larger cohort is needed to elucidate their prognostic value.

## Introduction

Lung cancer is the most common cause of cancer death worldwide [1]. The prognosis is best for patients with small tumors and no mediastinal or distant metastases. Patients with ipsilateral hilar lymph node metastases can receive surgery if otherwise fit. The TNM system is widely accepted for presurgical classification [2], and guides further treatment.

Many patients with small tumors and no apparent lymph-node metastases will still succumb to the disease. The five-year survival rate in patients with localized disease is 50% in females and 41% in males [3]. This indicates that a subset of patients with small tumors had metastatic spread prior to surgery, and that currently available methods for identifying such spread have failed. By identifying residual cancer cells, selected patients could receive adjuvant treatment in order to eradicate cancer cells not removed by surgery.

Several projects have aimed at finding markers to identify micrometastases and residual cancer cells, either as tumor cells in blood, lymph nodes (LN) or bone-marrow, or as RNA or proteins derived from cancer cells in blood, LNs or bone marrow [4], [5]. Common technologies for detecting metastases include reverse transcription polymerase chain reaction (RT-PCR), immunocytochemistry and immunohistochemistry. Tumor cell identification by the CellSearch system (based on immunomagnetic enrichment and immunofluorescense) or filtering procedures, have been performed both in a general lung cancer-population [5], [6] and in patients undergoing specific treatment in a clinical trial [7]. Quantitative RT-PCR analysis of lymph node lysates is thought to be a more sensitive technique compared with immunohistochemistry, and this method allows for investigation of entire LNs rather than only selected sections. Blood samples can also be assessed by RT-PCR to detect metastatic disease, and would be the least invasive of the methods for identifying metastatic cells.

Several different proteins and transcripts have been investigated as tumor cell markers in blood and LNs. The mRNAs for epithelial-specific cytokeratin (CK) 19 and 7 have been suggested as markers of microscopic lymphatic spread [8]. Expression of *SFTPB, TACSTD1*, and *PVA* have shown promising concurrence with lymph node metastases [9], and *CEACAM5* and *PLUNC* expression in lymph nodes were evaluated by RT-PCR, revealing a correlation with survival [10]. *CEACAM5* mRNA levels in lymph nodes showed an association with survival in a Chinese study of NSCLC patients [11]. The results have diverged, both in terms of detection rates and clinical impact, possibly due to disparities in methodology and sample sizes.

In this study, we analyzed several putatively interesting markers in LNs and in peripheral blood (PB) from patients with early-stage lung cancer undergoing surgery. We compared expression of the different markers in the tumors, the LNs, and PB samples in relation to patient’s clinical characteristics.

## Materials and Methods

### Patients

Patients admitted to Oslo University Hospital - The National Hospital for surgical treatment of histologically verified non-small-cell lung cancer (NSCLC) were recruited prospectively to the study during the period 2009 to 2010. Tumors from patients were included in the biobank depending on study nurse availability, and approximately 53% of the total number of lung cancer patients surgically treated during this period were included. The baseline classification according to age, sex, smoking status, stage (TNM-7-classification) and histology is presented in Table 1. Median age was 66.5 years.

**Table 1:** Clinicopathological parameters according to molecular examination of regional LNs and peripheral blood samples.

		Molecular LN status			Molecular CTC status		
	All patients	Negative	Positive	P value	Negative	Positive	P value
	N = 55	N = 16	N = 39		N = 29	N = 26	
**Median age (years)**	67	67	68	0.51*	67	67	0.88*
**Median packyears**	33	34	29	0.37*	36	28	0.08*
**Gender**				0.38			0.59
Female	32 (58)	11 (69)	21 (54)		18 (62)	14 (54)	
Male	23 (42)	5 (31)	18 (46)		11 (38)	12 (46)	
**pT stage**				1			0.33
1	13	4 (25)	9 (23)		9 (31)	4 (15)	
2	32	9 (56)	23 (59)		14 (48)	18 (69)	
3	7	2 (13)	5 (13)		5 (17)	2 (8)	
4	3	1 (6)	2 (5)		1 (3)	2 (8)	
**Median tumor diam. (cm)**	3.1	3.7	2.6	0.15*	2.7	3.2	0.24*
**Histology**				0.004			0.97
Adenocarcinoma	34 (62)	5 (31)	29 (74)		18 (62)	16 (62)	
Squamous cell carcinoma	13 (24)	7 (44)	6 (15)		7 (24)	6 (23)	
Other	8 (15)	4 (25)	4 (10)		4 (14)	4 (15)	
**pN stage**				1			1
0	43 (78)	13 (81)	30 (77)		23 (79)	20 (77)	
1	12 (22)	3 (19)	9 (23)		6 (21)	6 (23)	
**Clinical stage**				0.96			0.87
Ia	16 (25)	4 (24)	12 (31)		8 (28)	8 (31)	
Ib	24 (44)	8 (47)	16 (41)		14 (48)	10 (38)	
II	10 (18)	4 (24)	7 (18)		5 (17)	5 (19)	
III	5 (9)	1 (6)	4 (10)		2 (7)	3 (12)	

* Mann-Whitney test.

### Ethics Statement

The project was approved by the Norwegian Radium Hospital Institutional Review Board and the Regional Ethics Committee South East (permit number: S-05307). Written informed consent was obtained from each participant.

### Samples

All tumor biopsies were taken from presumably vital tumor tissue. In smaller tumors without signs of necrosis, the specimens were taken from the central part of the tumor. In larger tumors with signs of central necrosis, the specimens were taken from more peripheral parts of the tumor. Efforts were made to take pure tumor tissue, without surrounding lung tissue.

The LN sampling in most cases was done according to the European Society of Thoracic Surgeons (ESTS) guidelines [12]. The specimens were mainly taken from the expected drainage area of the tumor. A few exceptions occurred in situations when the surgeon suspected pathology in other LN stations.

One to four hilar LNs were dissected from the surgical specimens of all patients, leaving half the node for routine pathological review and one half for molecular analyses. Both the tumor tissue and lymph nodes were snap-frozen in liquid nitrogen in the operating room, and stored at −80°C until RNA isolation. The tumor cell content in the tumor specimens was more than 70% in most samples.

Sixteen cancer-free LNs from 8 patients undergoing surgery for benign colon diseases and 6 LNs from 6 patients undergoing surgery for benign pulmonary diseases were collected as normal LN reference material.

For all patients 2.5 ml blood was collected in PAX-tubes before surgery for RNA preservation. The blood samples were drawn from a venous port, and the samples drawn for research were not the first; hence, epithelial cell contamination was unlikely. Peripheral blood samples were also obtained from 12 healthy controls.

### RNA Extraction

LNs and tumor tissue were homogenized and lysed, and RNA was extracted using TRIzol (Invitrogen).

For blood samples, PAXgene blood RNA tubes were thawed at room temperature overnight. Total RNA was isolated using the PAXgene Blood miRNA Isolation Kit, according to the manufacturer’s instructions. RNA quantity and purity was assessed using the NanoDrop ND-1000 spectrophotometer (ThermoFisher Scientific, Wilmington, Delaware, USA). RNA quality was controlled by the Agilent 2100 Bioanalyzer (Agilent Technologies, Palo Alto, CA, USA).

### DNAse Treatment and Reverse Transcription

RNA was DNAse-treated by incubating 500 ng total RNA from each sample with 1 unit RQ1 RNAse-free DNAse (Promega) in a total volume of 10 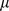
l 1× First Strand Synthesis buffer (Invitrogen) containing 10 units RNAseOUT RNAse inhibitor (Invitrogen). The reaction mixture was incubated at 37°C for 30 min and the DNAse inactivated by adding 1 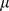
l RQ1 stop solution and incubating 10 minutes at 65°C. Complementary DNA was synthesized from the DNAse-treated RNA by M-MLV reverse transcriptase in a total volume of 20 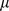
l according to the manufacturer’s protocol (Invitrogen).

### PCR Primers

At least one of the PCR primers in each primer pair was designed to span exon/exon boundaries or they were designed to bind to different exons. The identity of the evaluated marker transcripts and the primer sequences are listed in Table 2.

**Table 2:** Primer and MgCl_2_ concentrations in qPCR reactions.

Gene symbol	Gene name	Forward primer (5′->3′)	Reverse primer (5′->3′)	[Primer] (µM)	[MgCl_2_] (mM)
KRT19	Keratin 19	GATGAGCAGGTCCGAGGTTA	TCTTCCAAGGCAGCTTTCAT	0.3	2.00
CEACAM5	Carcinoembryonic antigen-related cell adhesion molecule 5	GGGACCTATGCCTGTTTTGTCTC	GAGCAACCCCAACCAGCAC	0.2	1.25
EPCAM	Epithelial cell adhesion molecule	CGCAGCTCAGGAAGAATGTG	TGAAGTACACTGGCATTGACG	0.3	1.25
DSG3	Desmoglein 3	GGCAAAAACGTGAATGGGTGA	GGGTTGCTTGGTAATCTGAAGTA	0.3	1.75
SFTPA	Surfactant protein A	TTGGAGGCAGAGACCCAAGCAG	GGCTCCAAGAAATCAGCGACCC	0.3	1.25
SFTPB	Surfactant protein B	GTCCAGCCCTCTCCAGTGTATC	GCCCGTCTCACTTGGCTTTTC	0.3	2.00
SFTPC	Surfactant protein C	AGCAAAGAGGTCCTGATGGA	ACAATCACCACGACGATGAG	0.3	1.25
BCR	Breakpoint cluster region	GCTCTATGGGTTTCTGAATG	AAATACCCAAAGGAATCCAC	0.15	2.00

### Quantitative PCR

PCR amplifications were performed with the qPCR SYBR Green Core kit (Eurogentec) according to the manufacturer’s recommendations. Reverse transcribed RNA (20 ng) was amplified in a total volume of 25 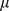
l containing 1× reaction buffer, 0.2 mM dNTP, 0,75 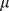
l 1∶200 SYBR Green I diluted in DMSO and MgCl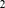, forward and reverse primer concentrations as shown in Table 2. Thermocycling and real-time fluorescence measurements were performed in an Mx3000P real-time PCR instrument (Stratagene), with an activation step of 10 min at 95°C followed by 40 cycles of 30 seconds at 95°C and 60 seconds at 60°C. Subsequently, the PCR products were analyzed by melting curves. All melting curves revealed well-defined peaks with the expected melting temperatures, confirming the specificity of the primers under the reaction conditions. Amplicon identities were also confirmed by sequencing. Reaction set-up, template addition and thermocycling were performed in in three separate, dedicated rooms. Controls containing no template were included in every run to monitor potential contamination.

Relative levels of each marker mRNA were determined by normalization against the BCR reference transcript and a calibrator sample included in every run, using the 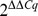 model [13], [14]. The calibrator sample was made by mixing RNA from the NCI-H441 (European Collection of Cell Cultures) cell line (50%) and two NSCLC tumors (25% each), chosen because of high levels of all potential markers. The reproducibility of the assays was determined by measuring the same reference sample in five successive experiments. The coefficients of variance determined were 7.5%, 9.6%, 6.1%, 7.3%, and 8.1% for the *CK19*, *CEACAM5, DSG-3, SFTPA*, and *SFTPC* assays, respectively. Threshold levels for positivity of each marker in blood and lymph were set to the highest levels in normal LNs and PB samples.

The real-time PCR quantifications were performed by two persons (G.S. and O.N.), who were blinded to the characteristics of the patients and primary tumors.

### Bioinformatic Marker Searches

Expressed sequence tag libraries (EST) and serial analysis of gene expression (SAGE) libraries were searched for candidate markers by the cDNA and SAGE digital gene expression displayer (DGED) tools at the Cancer Gene Anatomy Project (CGAP) web page (www.ncbi.nih.gov/cgap). In detail, all available EST libraries from normal adult lung tissue and lung cancers (pool A) were compared with libraries from normal LNs and normal peripheral blood mononuclear cells (pool B). The DGED tool produced a scoring list of genes ordered according to expression level differences between the two library pools, computed for each transcript as the ratio of sequences in pool A versus pool B. The SAGE DGED searches were done similarly. Transcripts residing in the top of both high-score lists (EST and SAGE DGED) were chosen for further characterization. High levels in many of the pool A libraries were preferred to extremely high levels in a limited number of them.

### Statistical Analysis

mRNA levels were not normally distributed and were compared using the Mann-Whitney U test. Categorical data were compared using Fisher’s exact test. Principal component analysis [15] was done by the *prcomp* function in R [16], scaling the variables to have unit variance before the analysis. Two-sided statistical tests were performed and p-values P
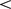
0.05 were considered statistically significant. All computations were done with the R software package (www.r-project.org) version 2.13.1.

## Results

### Selection and Validation of Candidate Markers

We systematically searched expressed sequence tag (EST) and serial analysis of gene expression (SAGE) libraries for mRNAs that were potential markers of tumor cells in lymph nodes (LNs) and peripheral blood (PB) from NSCLC patients. Candidate markers were scored according to the expression level difference between lung cancers and normal LNs/PB. From the lists of transcripts with highest scores, we selected keratin 19 (*KRT19*), carcinoembryonic antigen-related cell adhesion molecule (*CEACAM5*), epithelial cell adhesion molecule (*EPCAM*), surfactant proteins A (*SFTPA*) and C (*SFTPC*) for further validation as candidate markers. *KRT19, CEACAM5* and *EPCAM* mRNA had been reported previously as promising tumor cell markers in LNs from NSCLC patients [17], whereas *SFTPA* and *SFTPC* were novel genes in this context. Based on previous literature, we also selected desmoglein 3 *DSG3* and surfactant protein B (*SFTPB*) mRNA for further validation [17]. Surfactant proteins are essential components of the pulmonary surfactant fluid, which is important for the function and homeostatis of the lung alveoles. SFTPA is primarily involved in the defense against respiratory pathogens [18], whereas SFTPB and SFTPC maintain the accurate condition of the lipid surfactant film [19].

**Figure 1:**
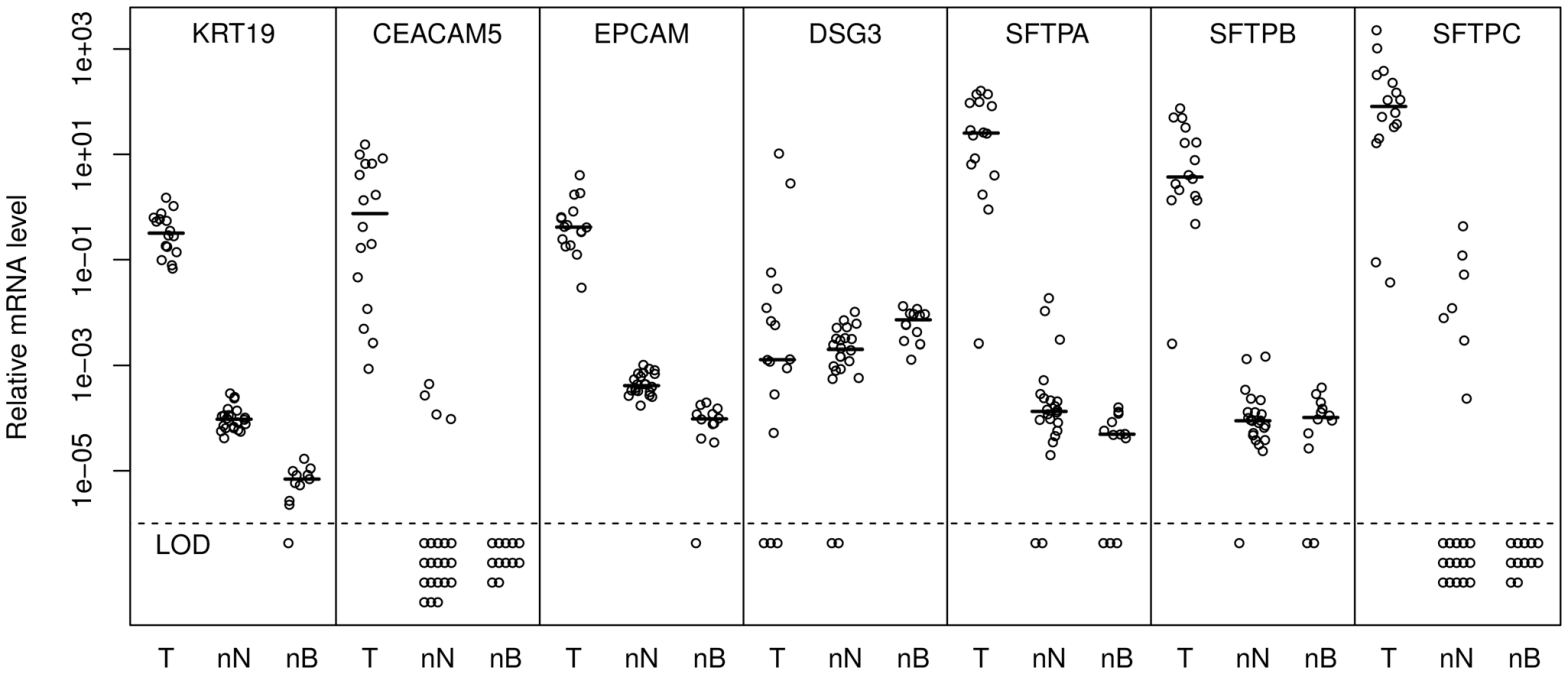
Relative marker levels in non-small cell lung cancer (NSCLC) tumors (T), normal LNs (nN) and peripheral blood samples (nB). Median values are indicated by short horizontal lines, whereas samples with levels below the limit of detection (LOD) are indicated below the dashed horizontal line. The levels of the different markers are relative to a calibrator sample and not directly comparable.

As a first validation of the 7 candidate markers, we measured their levels in 16 NSCLC tumor biopsies, 22 cancer-free LNs and 12 normal control PB samples by quantitative RT-PCR (Figure 1). Except for *DSG3*, the levels of all markers were much higher in the tumors compared with the control LNs and PB (P
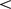
0.001). *CEACAM5* and *SFTPC* mRNA were undetectable in normal blood samples. Interestingly, the level of *SFTPA* mRNA in the 6 control LNs from lungs was significantly higher than in the control LNs from the colon mesentery (P = 0.002). A similar tendency, although not statistically significant, was also observed for *SFTPB* and *SFTPC*, but not for the remaining candidate markers.

We computed specificity indexes for each marker by dividing the median tumor level of each marker by the highest level in normal control LNs and PB samples (data not shown) [20]. The three highest specificity indexes were obtained for *SFTPB*, *CEACAM5*, and *SFTPA* (decreasing order) in LNs and for *SFTPA*, *KRT19*, and *SFTPB* in PB. Specificity indexes for *CEACAM5* and *SFTPC* in blood could not be computed because of undetectable marker levels in normal blood. We concluded that all evaluated candidates except *DSG3* seemed to have good potential as tumor cell markers in patient LNs and PB samples, according to expression level differences between tumors and the sample type of interest.

**Figure 2:**
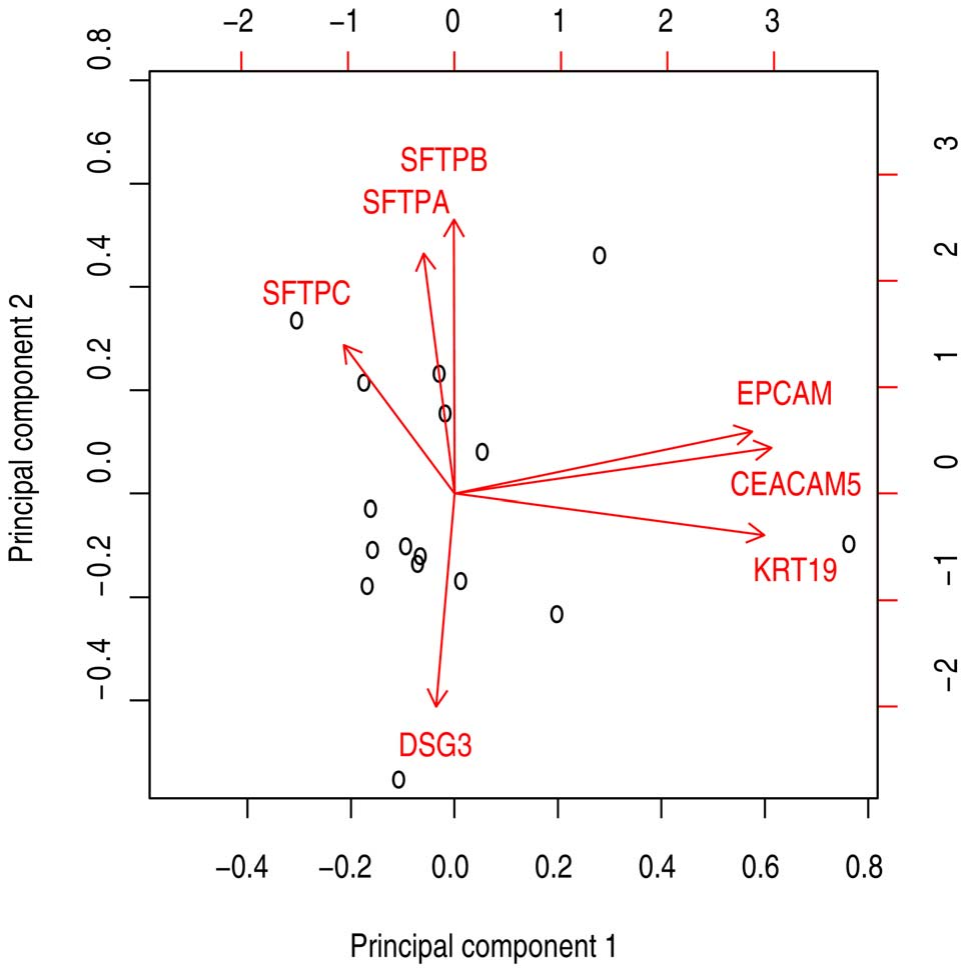
Biplot showing the results from principal component analysis of the 16 tumor samples. The black circles show the sample data projected onto the first and second principal components. The red arrows shows the old variable axes projected unto the principal components.

Specific epithelial mRNAs may be downregulated in subsets of tumors, reducing their utility as metastasis markers in the corresponding patients. Accordingly, we performed principal component analysis of marker levels in the 16 examined NSCLC tumors to identify covariations. The two first principal components explained 62% of the variance in the dataset. A biplot of the original variables (relative mRNA concentrations) and tumor samples projected onto the two first principal components demonstrated that *SFTPA*, *SFTPB* and *SFTPC* mRNA levels were correlated with each other (arrows pointing in the same direction), whereas *EPCAM* and *CEACAM5* and *KRT19* mRNA also covariated (figure 2). To choose a set of candidate markers optimally covering the spectrum of NSCLC cancers, we selected two markers from each of these covariation groups for further validation in addition to *DSG3*, which seemed to have an independent primary tumor expression pattern. The resulting marker panel consisted of *KRT19*, *CEACAM5*, *DSG3*, *SFTPA*, and *SFTPC* mRNA.

### Marker Levels in Tumors, LNs, and Blood Samples from NSCLC Patients

**Figure 3:**
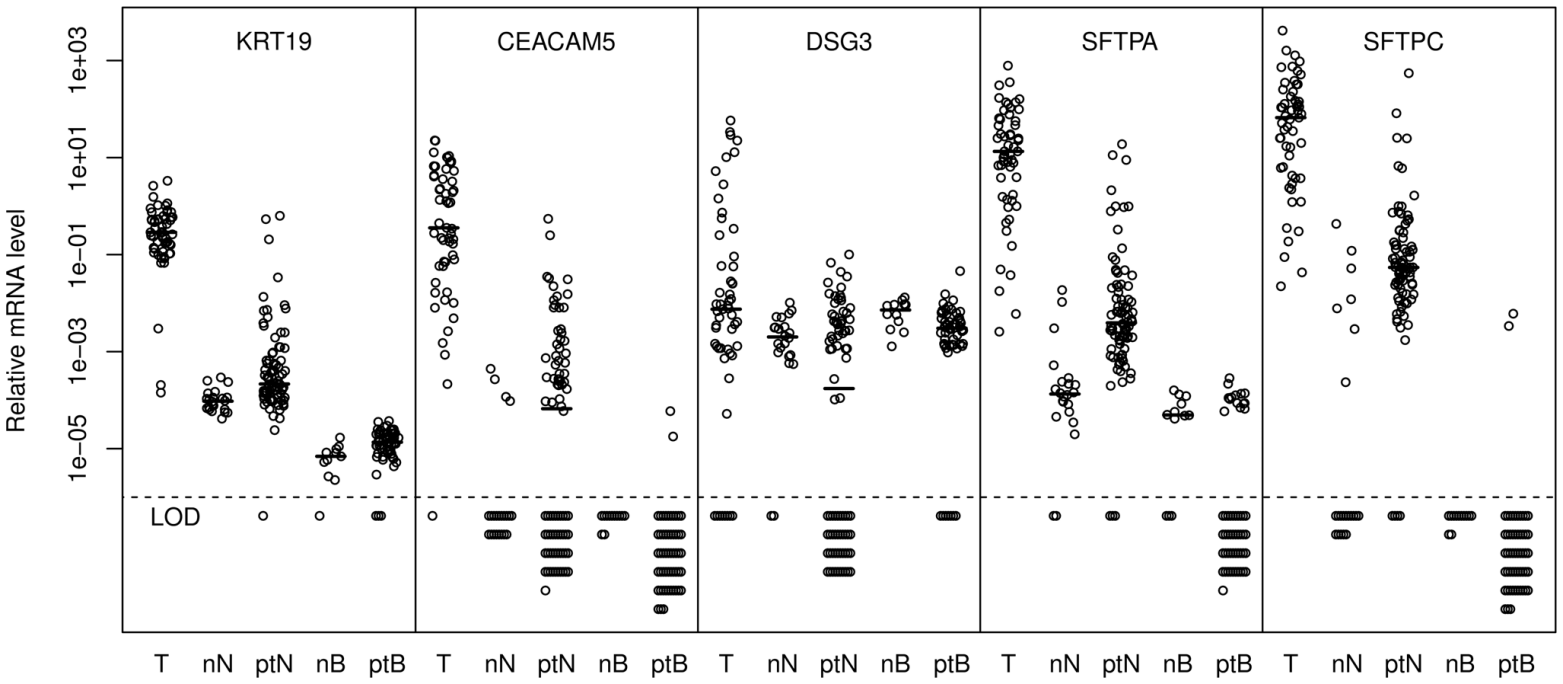
Marker levels in non-small cell lung cancer (NSCLC) tumors (T), normal LNs (nN), patient LNs (ptN), normal blood (nB) and patient blood (ptB). Median values are indicated by short horizontal lines, whereas samples with levels below the limit of detection (LOD) are indicated below the dashed horizontal line. The levels of the different markers are relative to a calibrator sample and are not directly comparable.

To further validate the 5 markers in the refined panel, we determined their relative levels in tumors (including the 16 from the initial validation), regional LNs, and PB samples from 55 NSCLC patients undergoing surgical treatment (Figure 3). A total of 84 LNs from the 55 patients were examined (mean 1.5 LN/patient, range 1–3). Some of the patients’ LNs and PB samples had elevated levels compared with the normal controls. However, marker levels in LNs retrieved from patients with positive node status (pN+) according to routine histological assessment were not significantly different from the other LNs, although there were clear trends for some of the markers (data not shown). We used the highest normal level of each marker as a threshold to define pathology in LNs and PB samples, since elevated levels most likely were due to the presence of tumor cells. Based on these thresholds, we determined the number of patients positive for each tumor cell marker in LNs and PB samples (Table 3). In total, 39 (71%) of the 55 patients were positive for at least one marker in the examined LNs, whereas 26 (47%) of the patients had positive PB samples. For LNs, all five markers contributed substantially to the identification of patients with molecular evidence of LN metastases. In PB samples, *KRT19* mRNA played a predominant role in identifying potential circulating tumor cells. Considerable overlap between the different markers was observed. There was no statistically significant association between LN and PB marker status.

**Table 3:** Number of patients with LNs and PB sampes positive for our 5 marker panel.

	Lymph nodes (%)	Blood (%)
**KRT19**	30 (55)	21 (38)
**CEACAM5**	20 (36)	2 (4)
**DSG3**	9 (16)	2 (4)
**SFTPA**	19 (35)	2 (4)
**SFTPC**	15 (27)	2 (4)
**At least 1**	39 (71)	26 (47)
**At least 2**	23 (42)	3 (5)
**At least 3**	18 (33)	0 (0)

### Comparison with Clinicopathological Data

Molecularly determined LN and PB tumor cell status and clinicopathological patient data (Table 1) were compared, but only one statistically significant association was identified. A significantly higher number of LN-positive patients had adenocarcinomas compared with other histological tumor types (P = 0.004). We tested whether this finding was related to the primary tumor levels of the individual markers, and found that *SFTPC* levels were significantly higher in adenocarcinomas than in other tumor subtypes (P = 0.005). However, *SFTPA*, *CEACAM5* and *DSG3* exhibited similar trends, with borderline significance (P = 0.06, P = 0.07, and P = 0.09, respectively).

**Figure 4:**
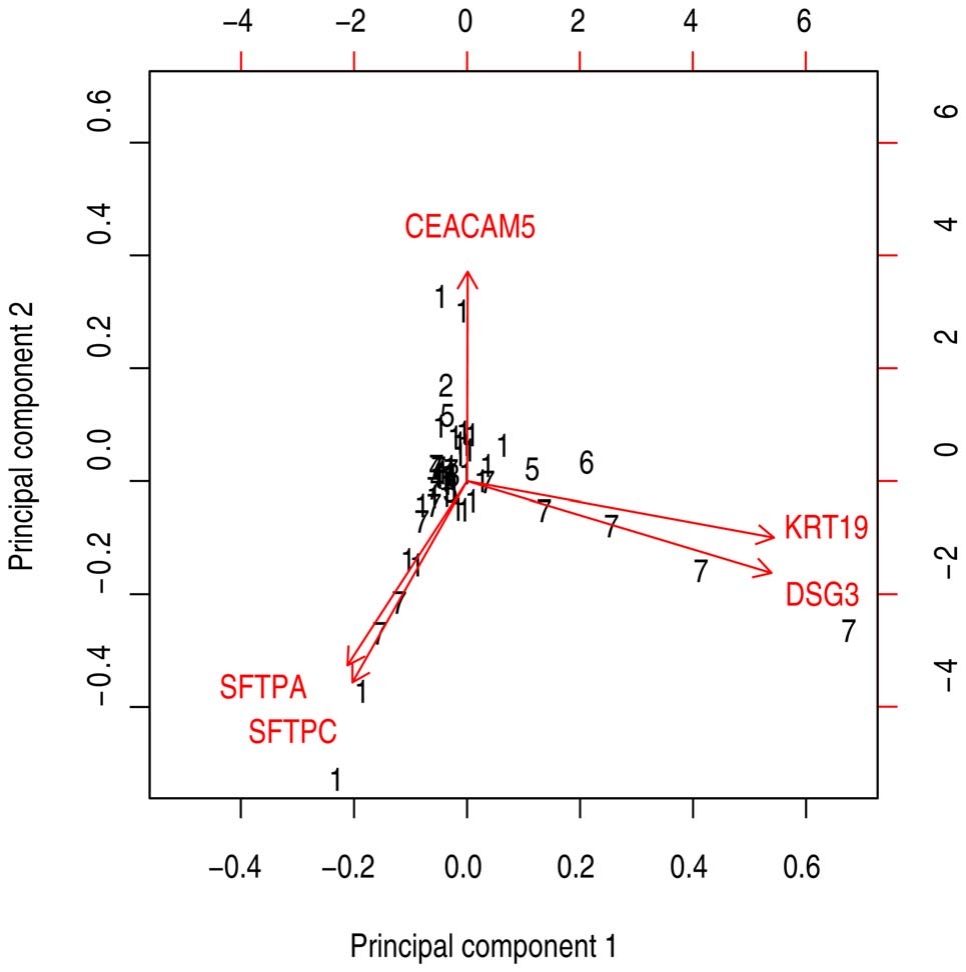
Biplot showing principal component analysis of *CK19*, *CEACAM5*, *DSG3*, *SFTPA* and *SFTPC* mRNA level in the 55 primary tumor biopsies. Black numbers indicate histology type (1 = adenocarcinoma, 2 = adenosquamous carcinoma, 3 = bronchioloalveolar carcinoma, 4 = carcinoid, 5 = large cell carcinoma, 6 = small cell carcinoma, 7 = squamous cell carcinoma).

To further investigate the relationship between primary tumor levels of each marker and histology subtype information, principal component analysis was performed (Figure 4). The resulting biplot showed that primary *SFTPA* and *SFTPC* levels were correlated, as well as *KRT19* and *DSG3* levels. Squamous cell carcinomas seemed to have high levels of both these marker groups, but not of *CEACAM5* mRNA. Mann-Whitney U tests confirmed significantly lower *CEACAM5* mRNA levels and higher *DSG3* mRNA levels in squaumous cell carcinomas (P = 0.003 and P = 0.002, respectively).

## Discussion

To investigate the clinical significance of tumor cell dissemination to regional LNs and PB in NSCLC patients, optimal detection methods are required. In our study we chose an indirect detection approach, employing epithelial-specific transcripts as surrogate markers for tumor cells. Accordingly, several promising markers for tumor cells in regional LNs and PB were identified and evaluated in the present study. Thorough validation in clinical samples revealed that *KRT19*, *CEACAM5*, *SFTPA*, *SFTPC* and *DSG3* were promising markers for tumor cells in LNs and PB from NSCLC patients. The *KRT19*, *CEACAM5* and *DSG3* markers have been reported previously [9], whereas *SFTPA* and *SFTPC* were novel in this context.

Based on the results from our validations and the principal component analysis of primary tumor levels shown in Figure 4, we suggest using a multimarker panel consisting of *KRT19*, *CEACAM5* and *SFTPA*. These three markers represent the three groups in the biplot analysis, suggesting that all 55 tumors in our validation cohort had high levels of at least one marker. All markers were found at very low levels in normal LNs and PB, whereas all except *DSG3* were expressed at high levels in most tumors. *DSG3* was expressed at high levels in most squamous cell carcinomas (Figure 4 and [9]), but the same was also true for *KRT19*. Because *KRT19* was also ubiquitous in other histological subtypes, and had a larger expression level differences between tumors, LNs, and PB, we favor this marker from the *KRT19*/*DSG3* group. The suggested multimarker panel needs to be investigated for clinical impact in future studies.

All markers evaluated in the present study were related to an epithelial phenotype, as a consequence of our inital search criteria. Recent data suggest that some CTCs undergo an epithelial-to-mesenchymal transition (EMT), which may facilitate their migration and ability to invade other organs [21], [22]. This transition is to some extent associated with a downregulation of epithelial genes, which means that affected CTCs will be more difficult to detect by assays relying on epithelial transcripts and proteins. Despite this, most presently available CTC enrichment and detection methods are based on epithelial markers [23], [24]. To reduce the problem, we suggest using a combination of several epithelial markers, like the multimarker assay suggested in the present paper. Such assays will be less vulnerable to downregulation of specific epithelial transcripts than single marker assays.

We expected higher positivity rates of our molecular markers in the LNs from patients with LN metastases identified by routine examination of all retrieved nodes (pN1), compared with node-negative patients (pN0). This expectation was based both on our previous molecular analyses of sentinel LNs from colon cancer patients [25], [26] and on previous reports of tumor cell dissemination in NSCLC patients [4], [27]. However, we did not observe any significant association between pN stage and our molecular LN analysis in the present study. One explanation for this may be the low number of nodes analyzed from each patient in our study (mean 1.5), which increased the relative probability of the presence of metastases in nodes not analyzed. To clarify this question it would be interesting to compare routine histological analysis of single LNs to our molecular analyses. However, histologically determined metastasis status for single nodes was not available in this study. On the other hand, nine of the 12 node-positive patients had LNs positive by our markers, which is acceptable taking the number of LNs analyzed into account. The main reason for the lack of statistical significance seems to be the high number of positive findings in otherwise node-negative patients, which may be due to occult metastases.

Similarly, we observed no significant association between clinical stage and circulating tumor cell (CTC) status. This contrasts with the study of Krebs et al, in which significantly more CTC positive stage IV lung cancer patients were found compared with those with stage III cancer [6]. Our study included very few stage III and no stage IV patients, making a direct comparison difficult. The number of CTCs is expected to be lower in early stage cancers, as observed in breast cancer [23]. Furthermore, Krebs et al. used the CellSearch system to detect CTCs, whereas we used real-time quantitative RT-PCR, also reducing the comparability. Moreover, the marker levels in our blood samples were barely above the detection limits (Figure 3), especially in the case of *CEACAM5* and SFPTC. Because of potentially low reproducibility near the detection limit, we reanalyzed all *CEACAM5* and *SFTPC* positive blood samples for confirmation (data not shown). The low marker levels probably corresponded to rather low CTC numbers. This is consistent with observations in early breast cancer patients [28]. In principle, detection of 1–2 CTCs is prone to low reproducibility. The CellSearch system is based on 7.5 ml blood samples. The blood sample volume in our study was limited to 2.5 ml, further reducing the likelihood of detecting CTCs.

The availability of hilar LNs from patients without cancer was low. Hence, we also analyzed 16 LNs from the colonic mesentery as normal reference material. It may be argued that mesenteric LNs are not strictly comparable with LNs from lungs. Accordingly, we did indeed observe higher levels of the surfactant protein mRNAs in the six mediastinal LNs. A simple explanation for this could be that the mediastinal LNs were contaminated by epithelial cells from the lungs, either through surgical handling or the normal physiological activity of the LNs. However, the fact that the other markers had similar levels in both LN groups seems to oppose that explanation. On the other hand, the surfactant mRNAs were not present in colonic epithelium. Nevertheless, because we used the highest marker levels in the control group as a threshold for positivity, the mediastinal LNs determined the threshold for the *SFTPA*, *SFTPB* and *SFTPC* markers.

We found no association between the LN and PB sample levels of our markers. This could be due to different routes of metastatic spread, as some tumors spread through the lymphatic system while others spread through the blood. High LN levels of epithelial-specific mRNA are thought to represent tumor cells with metastatic potential. However, such levels can represent cells originating from the tumor but that are in the process of being eradicated by the immune-system, or cell debris. Such discrimination could not be determined in this study, and the clinical impact needs to be evaluated in future studies.

No significant difference was identified in positivity rates between different cancer stages, or between men and women. More patients with adenocarcinomas had high tumor levels of some of the examined markers, compared with those with squamous cell carcinomas, but this should be interpreteded cautiously owing to the small sample size.

Clinicians need improvements in how to stratify patients for adjuvant therapy. Our gold standard, the TNM-classification system, does not provide satisfactory and accurate estimates of survival rates. This indicates that patients that could benefit from adjuvant treatment will not be offered such, whereas some patients cured by surgery alone receive unnecessary adjuvant therapy. Improved discrimination between patients with residual tumor cells, potentially in need of additional therapy, and those without this need, would benefit patients.

In conclusion, data supporting the clinical relevance of occult lymph node metastases and circulating tumor cells in lung cancer patients are emerging [5], [29]. Prediction of outcome and treatment response are among the potential clinical applications. In the present study, we identified a panel of promising tumor cell markers in LNs and PB samples from patients with early stage lung cancer. Further characterization is required to clarify the clinical impact of our findings and to identify new targets for improved risk prevention and tailoring of therapy.
